# Bats as an Important Source of Antimicrobial-Resistant Bacteria: A Systematic Review

**DOI:** 10.3390/antibiotics14010010

**Published:** 2024-12-26

**Authors:** Julio D. Soto-López, Manuel Diego-del Olmo, Pedro Fernández-Soto, Antonio Muro

**Affiliations:** Infectious and Tropical Diseases Research Group (e-INTRO), Biomedical Research Institute of Salamanca, Research Centre for Tropical Diseases at the University of Salamanca (IBSAL-CIETUS), Faculty of Pharmacy, University of Salamanca, 37008 Salamanca, Spain; jdjuliosoto@usal.es (J.D.S.-L.); manueldiego@usal.es (M.D.-d.O.); ama@usal.es (A.M.)

**Keywords:** bats, antibiotics, antimicrobial-resistant bacteria, zoonotic, public health, one health

## Abstract

**Background/Objectives**: Bats are the second-largest known order of mammals, accounting for about twenty percent of the species described to date. This group has special importance in health and epidemiology because they are considered hosts of a wide range of antimicrobial-resistant human pathogens. Over the past few decades, the emergence of pathogenic bacteria resistant to antimicrobials has been a growing threat to public health, especially given its repercussions such as deaths associated with antimicrobial resistance and economic losses in the healthcare sector. **Results**: The diversity of antimicrobial-resistant bacteria, the different methodologies in numeric analysis, and the variety of antibiotics reported in this review make it difficult to establish the scope of the effect of bats on the antimicrobial resistance crisis. **Methods**: In this systematic review, we focus on the existence of antibiotic-resistant bacteria associated with bats and summarize the main findings of studies conducted on the topic to date. **Conclusions**: Surveillance is essential to control the emergence of resistant bacteria related to bats, which could eventually affect humans, as this is a problem of a ‘One Health’ nature, with effects on human, animal, and environmental health.

## 1. Introduction

Bats (order Chiroptera) constitute the second-largest group of mammals and account for about 20% of all species described to date [1,2]. They are distributed worldwide, except in polar regions, deserts, and a few islands [2], and are known to be associated with antibiotic-resistant bacteria [3]. Several ecological and human factors contribute to the emergence of antimicrobial resistance (AMR) in bats, making them a key focus of One Health studies.

One major factor driving AMR emergence in bats is their interaction with diverse wildlife and environmental settings, which facilitates bacterial transmission. Habitat loss and urban growth reduce the natural roosts of bats and bring them closer to human communities, causing stress that leads to a weakening of their immune system [4]. This increases the likelihood of pathogen transmission among bats and directly affects the epidemiology of various diseases among the human population [5]. In addition, although bats are not primary agents in AMR dynamics, antimicrobial use in agriculture and veterinary practices may expose bats to resistant bacterial strains through contact with livestock and crops due to their feeding habits [3].

Bats have been shown to serve as vectors for transmitting certain AMR strains to humans [6]. Additionally, due to factors like their asymptomatic reservoir status, longevity, torpor, and migration habits, bats are efficient hosts for various pathogens, which they may disperse across a wide geographic area [7,8,9]. These characteristics should make bats a significant focus of study in human health dynamics, particularly regarding their interactions with antibiotic-resistant bacteria.

Given their broad distribution and unique ecological traits, bats interact with several microbial communities for which resistance to various antibiotics has been reported, such as chlamydia-like organisms (CLOs), which share an intracellular lifestyle and a biphasic development cycle with members of the genus *Chlamydia* [10]. Some CLOs have been associated with potential human health problems, such as adverse pregnancy outcomes (*Waddlia chondrophila*) or respiratory infections (*Simkania negevensis*) [11].

As for other bacteria potentially pathogenic to humans associated with bats, the bacteria *Corynebacterium*, causing diphtheria, skin infections, and septicemia, and *Mycobacterium*, the etiologic agent of tuberculosis, have been found in *Rhinolophus monoceros* bats [12]. Other species have also been isolated from bats, such as *Bacillus cereus* and *B. anthracis*, well-known bacterial pathogens that exhibit virulent features such as hemolytic and proteolytic activities, and *Pseudomonas stutzeri*, an opportunistic human pathogen [12]. *Clostridium difficile*, an anaerobic bacterium capable of inducing severe disease in humans requiring hospitalization [13], has been isolated from the organs, blood, and feces of several species of bats.

The presence of pathogenic bacteria in bats raises several important public health concerns due to their potential to acquire and disseminate antimicrobial resistance. There are several mechanisms that allow these bat-associated bacteria to develop AMR [14] among them (1) limiting the entry of drugs into bacterial cells by preventing the internal accumulation of antibiotics through porin channels and efflux pumps; (2) modifying antibiotic target molecules by altering their binding through changes in 30S or 50S ribosomal subunits, penicillin-binding proteins, DNA gyrase, topoisomerase enzymes, or D-alanyl-D-alanine [15,16,17]; (3) enzymatic antibiotic inactivation through beta-lactamase enzymes, aminoglycoside-modifying enzymes and chloramphenicol-acetyl-transferase enzymes that break down, prevent the attachment of, or disable the binding of the antibiotic molecules [16,18]; (4) random genetic mutations, which occur naturally due to modulations in gene expression in response to environmental changes [19]. If a mutation provides a survival advantage in the presence of an antimicrobial, it is more likely to be transmitted to subsequent generations [14]. In such a case, AMR occurs through horizontal gene transfer (HGT), which involves the transference of genetic material (including resistance genes) among bacteria, even among different species, allowing the transfer of genetic information associated with antimicrobial resistance (transformation, transduction, and conjugation) [19,20,21]. (5) Adaptations to environmental stress can also cause AMR in bat-associated bacteria. In nature, bacteria are confronted with various environmental stressors, including natural antimicrobial substances [19]. For example, bacteria have developed an adaptation that consists of the formation of biofilms as communities of cells enclosed in a protective matrix. This enhances resistance to antimicrobials by physically protecting them from the effects of antimicrobial agents and by facilitating the exchange of resistance genes in the bacterial community within the biofilm. There is also the production of antibiotics by fungi naturally as a defense mechanism. In response, other microorganisms may develop resistance mechanisms to these natural antimicrobials, which can then confer cross-resistance to clinically relevant antibiotics [19,20,21].

It is important to note that the development of AMR in nature is a dynamic and continuous process influenced by numerous ecological and selective pressures. Human activities, such as overuse or misuse of antimicrobials in healthcare, agriculture, aquaculture, animal husbandry, and other sectors, can accelerate the emergence and spread of AMR in natural environments.

AMR in bats has profound implications for public health and reflects the complex interplay of ecological pressures captured by the ‘One Health’ framework. With an estimated 4.95 million (3.62–6.57) AMR-associated deaths in 2019 [22], the scope of this pandemic is broad and potentially devastating. In addition, AMR is responsible for an estimated 192,000 (146,000–248,000) increased disability-adjusted life years (DALYs) as well as significant healthcare economic losses [22]. If unchecked, AMR could cause even more deaths. Although criticized [23], the Review on Antimicrobial Resistance, commissioned by the British Government, stated that AMR could cause 10 million deaths and increase global healthcare costs by up to USD 1 trillion per year by 2050 [24]. Despite this, it is clear and there is a consensus among the scientific community that coordinated global action is needed to tackle AMR [6,22,25,26].

Due to the importance of AMR bacteria associated with bats and their potential impact on public health dynamics, our aim was to conduct a systematic review of all reported cases of bat-associated AMR bacteria available to date.

## 2. Results

### 2.1. Synthesis of Results

**Figure 1:**
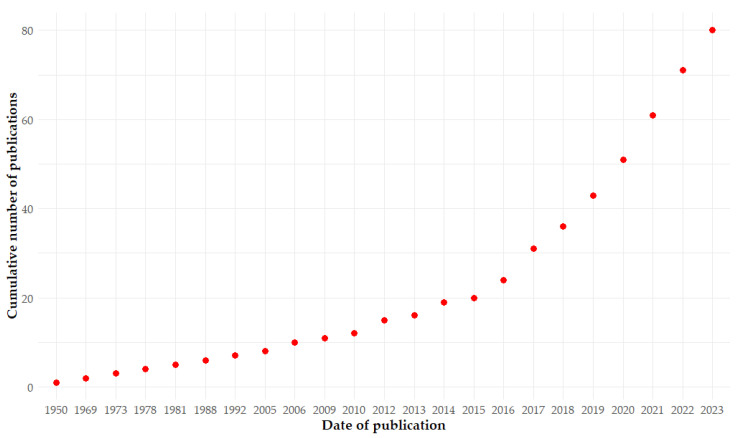
Graph showing the increase over time in the number of publications appearing in PubMed Central that mention the relationship between antibiotic resistance and bats. Search terms were ‘resistance + antimicrobial + bats’. Publications (total = 82) were identified using the PubMed R package version 0.0.3 on 1 October 2024 [27].

The number of publications mentioning antimicrobial resistance as well as bats followed a linear increase until 2015; thereafter, and to date, there has been an exponential increase with no signs of stabilization (Figure 1).

Bacterial resistance to antibiotics has been identified as one of the main threats to global public health. In recent years, the development of different technologies and research methods, such as massive sequencing, has enabled the detailed study of bat microbiota and AMR, which has allowed the isolation of bacteria resistant to several antimicrobials in different bat species (Supplementary Table S1).

### 2.2. Bats

Studies on antibiotic-resistant bacteria associated with bats have been conducted in Neotropical, Afrotropical, Indomalayan, and Palearctic species (Supplementary Table S1). The authors reported on bat species with AMR bacteria that show no clear pattern in terms of their feeding habits, as they are frugivorous, insectivorous, hematophagous, and nectarivores species. In terms of their association with humans, species show varying degrees of interaction with human-modified environments, including synanthropic species (which frequently inhabit human structures and may interact with activities such as agriculture or livestock) and wild species (which mainly inhabit forests, jungles, or caves away from urban environments and have minimal contact with humans).

Some of these species may pose a potential public health problem due to their closer contact with humans, especially those that take refuge in anthropogenic environments, which could increase the risk of zoonotic transmission.

### 2.3. Bacteria

According to their clinical relevance and involvement in human infections [28], the presence of the following bacteria has been described: (1) Bacteria of importance in nosocomial and community-acquired infections include *Escherichia coli* and *Shigella* sp., commonly associated with dysentery, urinary tract infections, bacteremia, and neonatal meningitis; *Klebsiella* spp. (*K. pneumoniae*, *K. oxytoca*, *K. ozanae*, and *K. aerogenes*) and *Acinetobacter baumannii* associated with nosocomial pneumonia; *Pseudomonas aeruginosa*, linked to infections in patients with cystic fibrosis, burns, and wounds; and *Staphylococcus aureus*, associated with skin infections, pneumonia, endocarditis, and bloodstream infections [29]. (2) Opportunistic bacteria such as *Staphylococcus* spp. (*S. epidermidis*, *S. saprophyticus*, *S. haemolyticus*, *S. warneri*, *S. xylosus*, *S. argenteus*, *S. schweitzeri*, *S. nepalensis*) linked to infections from medical devices and catheters; *Enterobacter* spp., *Proteus* spp., *Serratia marcescens*, *Serratia liquefaciens*, *Citrobacter* spp., *Providencia* spp., *Morganella* spp., *Stenotrophomonas maltophilia*, *Enterococcus* spp., and *Aerococcus* spp. associated with nosocomial infections, mainly urinary and respiratory tract infections; *Bacillus cereus*, linked to food poisoning, and *B. anthracis,* linked to anthrax; bacteria associated with opportunistic infections, mainly in immunocompromised patients, such as *Rahnella aquatilis*, *Arthrobacter* spp., *Kocuria* spp., *Moellerella* spp., *Pseudomonas* spp., *Mammaliicoccus sciuri*, *Achromobacter* spp., and *Kluyvera* spp.; and *Alcaligenes* spp., linked to nosocomial infections, mainly in patients with chronic lung diseases [30]. (3) Important zoonotic and environmental bacteria such as *Salmonella* spp. (*S. gallinarum*), associated with gastrointestinal infections and typhoid fever; *Waddlia malaysiensis* with zoonotic potential, implicated in respiratory and reproductive infections; and *Enterobacter agglomerans* and *Erwinia herbicola,* generally non-pathogenic to humans and found mainly in plants. They may occasionally cause opportunistic infections in immunocompromised individuals [31].

According to their classification and mechanism of action [14], AMR described in bat-associated bacteria (Supplementary Table S1) has been associated with the following:

- Natural penicillins (Pe: penicillin), aminopenicillins (A: amoxicillin; Ap: ampicillin), antipseudomonal penicillins (Pi: piperacillin; Ti: ticarcillin), beta-lactamase-resistant penicillins (O: oxacillin; M: Methicillin), and broad-spectrum penicillins (Tc: temocillin).
- First-generation cephalosporins (C: cephalexin), second-generation cephalosporins (Ce: cefoxitin), third-generation cephalosporins (Cx: cefotaxime; Cf: ceftazidime; Ct: ceftriaxone), and fourth-generation cephalosporins (Cp: cefepime).
- Carbapenems (I: imipenem; Er: ertapenem).
- Monobactams (Az: aztreonam).
- Aminoglycosides (Am: amikacin; Ge: gentamicin; K: kanamycin; Ne: netilmicin; S: streptomycin; To: tobramycin).
- Macrolides (E: erythromycin) and lincosamides (Cl: clindamycin; Li: lincomycin).
- Fluoroquinolones (Ci: ciprofloxacin; Le: levofloxacin; No: norfloxacin; Of: ofloxacin).
- Tetracyclines (Mi: minocycline; TE: tetracycline).
- Other antibiotics (Ch: chloramphenicol; Fu: fusidic acid; Fo: fosfomycin; Mu: mupirocin; N: nalidixic acid; Sx: sulfamethoxazole/trimethoprim; Tm: temocillin; Va: vancomycin).

The geographical distribution of antimicrobial-resistant bacteria associated with bats clearly shows that most publications on the subject are from Australia and Nigeria, followed by Brazil, Peru, Indonesia, Poland, and Slovakia (Table 1).

### 2.4. Identification of AMR

The identification of AMR in microorganisms is a process that consists, first, of determining the presence of antimicrobial-resistant bacterial colonies. Currently, AMR is identified by the disk diffusion method (also known as the Kirby–Bauer test), which is suitable for fast-growing microorganisms in the presence of different antibiotic concentrations [32].

The second part of this process involves the isolation of colonies and their taxonomic identification by phenotypic [33] or molecular methods, such as matrix-assisted laser desorption/ionization–time of flight mass spectrometry (MALDI-TOF), and PCR amplification of reference genes, such as 16S or ITS. This step can also be performed by comparative genotypic analyses such as the amplification of DNA fragments surrounding rare restriction sites (ADSRRS fingerprinting), restriction endonuclease digestion of total genomic DNA, multiplex PCR identification, and sequencing (whole-genome sequencing or metagenomics) [32,34].

**Figure 2:**
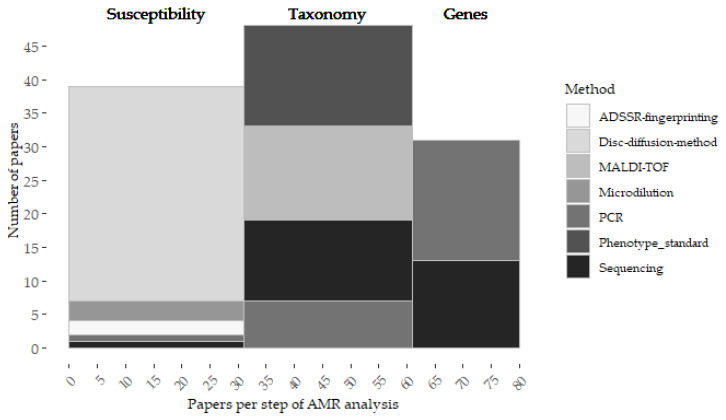
Methods of identification of susceptibility to antibiotics in bacteria associated with bats, taxonomy determination of bacteria, and antibiotic resistance gene analysis. ADSRRS fingerprinting: amplification of DNA fragments surrounding rare restriction sites; disk diffusion method: Kirby–Bauer’s antibiotic disk diffusion technique; MALDI-TOF: matrix-assisted laser desorption ionization–time of flight mass spectrometry; PCR: polymerase chain reaction; Phenotype_standard: classical biochemical methods (Gram staining, catalase, oxidase, indol, Methyl Red–Voges–Proskauer, and citrate) and by the API 20E system. Records from archives of PubMed Central^®^ and the Web of Science platform after filtration.

Despite the current development of ADSRRS fingerprinting, the identification of AMR in bat-associated microorganisms is mainly performed by the traditional disk diffusion test (31/36) (Figure 2). This test is simple and inexpensive, which may explain its use over the other techniques described.

The subsequent identification of molecules responsible for AMR can be performed through the amplification of specific genes or by sequencing to search for genes encoding for resistance, such as extended-spectrum β-lactamases (blaSHV, blaOXA, blaTEM, blaCTX-M), aminoglycosides (aph 3′-Ia and aph 3′-IIa, strA, aac 3-II, aac 3-III, aac 3-IV), tetracycline (tetA, tetB), phenicols (cmlA, floR, cat), sulfonamides (sul1, sul2, sul3), integrase genes (int1 and int2), and even genes that encode virulence factors, such as fimA (type 1 fimbriae), aer (aerobactin), papC (type P fimbriae), hly (α-hemolysin) and bfpA (type IV bundle-forming pili) [3,35].

Most studies analyzed aimed to identify bacteria at the species or genus level. In those 36 studies on their taxonomic identification, this was mostly addressed by MALDI-TOF and phenotypic standard techniques, although an increase in the use of sequencing to accomplish this task could be observed among the studies analyzed. It is important to note that MALDI-TOF is a precise and rapid technique that can be complemented by traditional phenotypic analyses, which explains its widespread use in microbiology laboratories. Despite this, the use of sequencing can improve the range of organisms that can be studied since the databases used for comparative purposes when sequencing are usually more comprehensive than those used when using MALDI-TOF.

Contrary to the taxonomic identification of the bacteria involved in AMR, the identification of genes encoding molecules associated with resistance is a process less commonly performed in studies focused on AMR bacteria associated with bats (19/36). However, the identification of the genes involved was performed in a high percentage of the studies analyzed, mainly through sequencing, followed by polymerase chain reaction and sequencing of benchmark makers. This step is crucial since it provides information on AMR mechanisms, in which direct intervention can be made to control antimicrobial-resistant microorganisms.

### 2.5. Statistical Analysis

We found that 35/36 studies in this review are descriptive ones, with only 20% including statistical analyses (Table 2). They evaluated differences in the presence of resistant bacteria or resistance genes among different bat species. They also examined variations in antimicrobial resistance (AMR) across bacterial species, antimicrobial agents, years, and locations. Additionally, they compare AMR in livestock, bats, and various human populations. The publications show significant differences (alfa 0.05) among antimicrobial agents, livestock against bats, and bacterial species. Mixed results were found between sites and years. It is probable that the distribution of resistant bacteria associated with bats may be wider than is currently known.

The statistical analyses of the publications included in this review were performed in a non-systematic way. Most of the included studies suffer from a lack of statistical power, lack of replications, different sizes and variability of the samples used to detect the effect, or contradictory data (for example, differences in prevalence between samples in different regions).

## 3. Discussion

Antimicrobial resistance is a growing global concern with several implications for public health, particularly in the context of zoonotic pathogens. Bats, as reservoirs of human pathogens, have raised attention due to their potential role in the dynamics of AMR. In this review, we examine the existing publications on AMR in bat-associated bacteria, highlighting classification according to human importance, geographic distribution, methods of identification, and statistical analysis.

We observed a growing interest in AMR in bacteria associated with bats in the last decade. From 2015, the number of publications on this topic showed an important increase, suggesting specific interest in AMR and bat-associated bacteria.

Early studies likely reflected a broader concern about AMR’s impact on human health across different organisms. However, the sharp rise in publications from 2016 onwards may indicate an increase in research funding and interest. The COVID-19 pandemic has significantly increased recognition of the importance of this issue and highlighted the need for a One Health approach to understanding zoonotic reservoirs.

Regionally, Australia has led in publications, driven by research groups studying *Pteropus poliocephalus* (a common bat species in this region) through collaborations with wildlife rehabilitation organizations. This prominence is likely supported by a strong interest in biodiversity conservation, public health funding, and a focus on zoonotic diseases. Nigeria ranks second, with research from institutions like Obafemi Awolowo University, addressing the public health implications of zoonotic reservoirs and the role of fecal contamination in public health.

Antimicrobial susceptibility testing in bat-associated bacteria has revealed widespread resistance to many commonly used antibiotics. Studies have concentrated on the six leading pathogens responsible for most deaths associated with AMR, including *E. coli*, *S. aureus*, *K. pneumoniae*, *S. pneumoniae*, *A. baumannii*, and *P. aeruginosa* [22].

Resistance has been detected across various countries, such as Italy, Nigeria, Brazil, Australia, Poland, the Republic of Congo, Peru, Trinidad and Tobago, China, Spain, Slovakia, Portugal, Algeria, Indonesia, Malaysia, Chile, Japan, Slovenia, and Gabon [3,35,36,43,44,45,46,47,48,49]. This indicates that the AMR in bat-associated bacteria is a global phenomenon.

Most of the publications describe AMR in bats to be due to the acquisition of AMR-related genes, the acquisition of entire transposons, and the transmission of resistant bacteria between different bat species [3,36,38,44,45,49,50]. This highlights the need for the early detection of resistant bacteria strains plus the monitoring and surveillance of bat populations in their environment.

The presence of AMR in bat-associated bacteria poses several challenges. Bats could indirectly contribute to resistant infections in humans, especially in regions where humans frequently interact with wildlife or bats inhabit urban areas. Exposure to antibiotics in agricultural and veterinary settings increases selection pressure. Antibiotics degrade in natural ecosystems, which provides a non-lethal dosage to bacteria, fostering the right environment for the adaptability of microorganisms [48] and promoting the survival of resistant strains. Also, the constant contact of bats with agricultural or livestock production areas brings antibiotic molecules into contact with bats, promoting bacterial dispersion across species.

Addressing these risks requires a One Health approach, with responsible use of antimicrobials in veterinary practices and agriculture to help minimize the selection pressures for resistant bacteria. Research is important to elucidate the specific mechanisms and pathways of resistance transmission. Long-term surveillance studies, together with genetic and ecological analyses, can provide valuable insights into the dynamics of antimicrobial resistance in bat populations.

The reviewed studies were largely descriptive, limiting the ability to distinguish patterns. However, they provide valuable baseline data for future research and for identifying cross-contamination patterns. However, due to the descriptive nature of these studies, it is not possible to make generalizable inferences or to establish or quantify causal relationships. Including more robust statistical analyses could help to identify trends and provide robustness to the conclusions of the studies.

To address this, future research should adopt standardized methodologies; harmonizing protocols across studies will improve comparability and strengthen conclusions. They must also integrate advanced statistical analyses to identify patterns and quantify the impact of specific variables on AMR dynamics.

Expanding surveillance programs of bat populations and their environments can enable the early detection of resistant strains. But also, promoting responsible antimicrobial use will reduce the misuse of antibiotics in agriculture and veterinary practices, minimizing selection pressures for resistance. Because AMR is an increasingly important global issue and given that addressing it from a One Health perspective is clearly advantageous, it is essential to standardize the studies carried out in wildlife to improve AMR management strategies [25,26].

## 4. Materials and Methods

### Eligibility Criteria and Information Sources

A systematic review was performed following PRISMA guidelines with articles selected through a double-blind peer review process; a third reviewer was consulted in cases of disagreement. All studies focusing on bat-associated antimicrobial-resistant bacteria were searched for on PubMed Central^®^ and Web of Science until 1 October 2024. The search terms used (search string) were “resistance”, “antimicrobial”, and “bats”, obtaining a total of 164 records. Records were downloaded from the databases using the corresponding search tool. After eliminating duplicate studies, 127 records remained.

Inclusion and exclusion criteria were applied, accepting only studies dealing with antimicrobial-resistant bacteria isolated from bats and excluding those analyzing viruses, bacteria of other species, records without bat taxonomy, non-accessible articles, review articles, book chapters, letters, editorial material, meeting abstracts, clinical trials, meta-analyses, and systematic reviews (29 excluded records). Study selection was performed by two reviewers independently, first analyzing titles and abstracts, and then the full text of articles that passed the first filter. Any disagreement was resolved by consulting a third reviewer.

**Figure 3:**
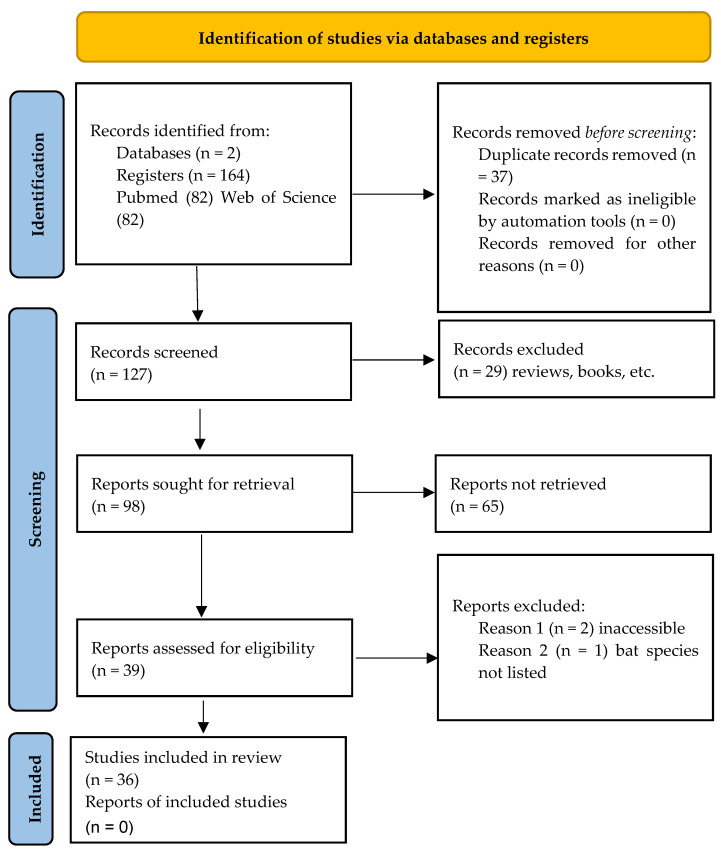
PRISMA 2020 flow chart. Only database and registry searches are indicated.

After this filtering process, a total of 36 studies were obtained that met the inclusion criteria (Figure 3). The extracted data were organized in tables, including information on the bat species, the bacteria isolated, and the type of resistance observed. Subsequently, the data were analyzed using the R statistical program, performing exploration analysis between bacterial resistance and variables such as bacterial species, host species, and host feeding habits.

Limitations of this review include the potential exclusion of relevant studies due to language barriers, as some authors from non-English-speaking countries may not publish in Western databases, as well as methodological variability among studies, which may affect the comparability of results and the deduction of conclusions.

## 5. Conclusions

Antimicrobial resistance is a global public health issue and its presence in non-human animal species, such as bat colonies, highlights the interconnectedness of ecosystems and the need for a ’One Health’ approach. Bats harbor numerous bacteria resistant to a variety of commonly used antibiotics, posing potential risks for zoonotic spillover events and challenging the management of infectious diseases. Addressing this problem requires collaboration between researchers, veterinarians, and public health officials.

Understanding antimicrobial resistance dynamics in bats is essential not only for identifying risk factors but also for designing effective prevention and control strategies. This includes reducing environmental exposure to antibiotics, enhancing surveillance systems in wildlife, and promoting responsible antimicrobial practices in agriculture and veterinary medicine.

Given the complexity and evolving nature of AMR in bats, addressing this issue requires robust interdisciplinary collaboration. Researchers, veterinarians, public health officials, and policymakers must work together to implement integrated solutions that mitigate risks at the human–animal–environment interface. This includes advancing genetic and ecological studies to understand resistance mechanisms, standardizing surveillance protocols to monitor trends effectively, and aligning public health policies with conservation strategies. Such efforts can mitigate the risk of AMR in public health.

## Figures and Tables

**Table 1:** Number of publications about AMR in bacteria associated with bats by country.

Country	Publications	Country	Publications	Country	Publications
Australia	6	Slovakia	2	Japan	1
Nigeria	5	Algeria	1	Malaysia	1
Brazil	3	Chile	1	Portugal	1
Congo	2	China	1	Slovenia	1
Indonesia	2	Gabon	1	Spain	1
Peru	2	India	1	Trinidad and Tobago	1
Poland	2	Italy	1		

**Table 2:** Statistical analysis conducted in publications on AMR in bacteria associated with bats.

Comparison	Test	Alfa	Results	Source
Prevalence of resistant *E. coli* to several antimicrobial agents	Chi-square (χ2) test	0.05	<0.05	[36]
Prevalence of ESBL-*E. coli* fecal carriage in bats and livestock	95% CI, Pearson’s chi-squared, Fisher’s exact test	0.05	5% [CI: 3–7%]; 20 out of 388—48% [CI: 40–56%] Pearson’s chi-squared test, *p* < 0.0001	[37]
Prevalence of ESBL-*E. coli* across years			10% in 2015, 4% 2017, and 4% 2018, Fisher’s exact test, *p* = 0.14	
Prevalence of ESBL-*E. coli* among five bat colonies			Fisher’s exact test, *p* = 0.76	
Sites with a significantly higher number of multidrug-resistant (MDR) strains	Chi-square (χ2) test, Bonferroni correction	0.05	*p* = 0.0033	[38]
Comparison of the number of MDR strains in bat species			*p* = 0.0083	
Resistance factor among bacterial isolates obtained from *Cynopterus* spp. and *Myotis muricola*: (a) *Morganella* sp. (b) *Proteus* sp. (c) *E. coli* (d) *Klebsiella* sp. (e) *Citrobacter* sp.	Chi-square (χ2) test or Fisher’s exact test (sample size dependent)	0.05	*p* < 0.05, *p* < 0.05, *p* > 0.05, *p* > 0.05, *p* > 0.05	[39]
Comparison of AMR patterns in *E. coli* from rectal swabs of *Cynopterus* sp. on two islands			*p* < 0.05	
Comparison of AMR patterns in *Klebsiella* from rectal swabs of *Cynopterus* sp. on two islands			*p* > 0.05	
Comparison of *Klebsiella* sp. isolates from *Cynopterus* sp. in terms of susceptibility to sulphamethoxazol on two islands			*p* < 0.05	
Comparison of AMR patterns of *Enterobacter spp.* from rectal swabs of *Cynopterus* sp. on the following: (a) Rakata (b) Panjang (c) Sertung			*p* < 0.05, *p* > 0.05, *p* > 0.05	
Difference in the mean of multiple AMR indices of *E. coli* isolates from two bat species	T-student	0.05	T = 0.702	[35]
Association between the proportion of *E. coli* isolates resistant to the following: (a) At least one antimicrobial agent. (b) Multiple drugs and bat species.	Chi-square (χ2) test		χ2 = 0.221, χ2 = 0.717	
Differences in prevalence between samples in different regions.	Chi-square (χ2) test, Fisher’s exact test (sample size dependent).	0.05	*p* = 0.07	[40]
Differences in prevalence between samples originating from urban or rural municipalities.			Chi = 0.13, *p*-value = 0.72	
Correlation between the presence/absence of AMR in bats and the following: (a) Municipality’s human population size. (b) Population density.	General linear mixed-effect model with binomial residual distribution		AIC = 249, estimate = −1.38 × 10^−3^, *p*-value = 0.16 AIC = 251, estimate = 0.01 × 10^−5^, *p*-value = 0.87	
Differences in intI1 and blaTEM in paired samplings collected from fire-affected (2021) and pre-fire bats (2018–2019) in the following: (a) Sydney (b) Adelaide	Fisher’s exact test	0.05	*p* > 0.05, *p* < 0.05	[41]
Differences in the expression of intI1 and blaTEM in in-care bats (Sydney 2020) compared to wild bats (Bega 2019–2020)			*p* < 0.05	
Differences in bats receiving antibiotics compared to those that did not receive antibiotics in the expression level of the following genes: (a) intl1 (b) blaTEM			*p* = 0.4007, *p* = 0.2455	
Explain observed variation in methicillin-resistant *Staphylococcus aureus* carriage in infected hosts	Generalized linear model	0.05	Estimate = 1.93, *p*-value = 0.009 AIC = 63.723, Std. Err = 0.73	[42]
Prevalence of *S. aureus* in the host community	Chi-square (χ2) test	0.05	*p* = 0.705	

CI: confidence interval; Std. Err: standard error.

## Data Availability

Data is contained within the article or Supplementary Materials.

## Supplementary Materials

The following supporting information can be downloaded at https://www.mdpi.com/article/10.3390/antibiotics14010010/s1: Table S1: Antibiotic-resistant bacteria in bats [35,51,52,53,54,55,56,57,58,59,60].
