# Supplementary material for: Bats as an Important Source of Antimicrobial-Resistant Bacteria: A Systematic Review
Source: Antibiotics (Basel). 2024 Dec 26;14(1):10. doi: 10.3390/antibiotics14010010 (PMC11761237; doi:10.3390/antibiotics14010010)
Supplement: Supplementary file 1 [file antibiotics-14-00010-s001.zip › antibiotics-3378993-supplementary.pdf]

Supplementary Table S1. Antibiotic-resistant bacteria in bats

Bats	Bacteria	Antibiotic																																									
		A	Am	Ap	AZ	C	Ce	Cf	Ch	Ci	Cl	Cp	Ct	Cx	Do	E	Er	Fo	Fu	Ge	I	K	Le	Li	M	Mi	Mu	N	Ne	No	O	Of	Pe	Pi	S	Sx	Tc	TE	Ti	Tm	To	Va	So
Anoura caudifer	*Klebsiella oxytoca*	-	-	+		-		-	-	-			-		-					-	-																						49
Anoura geoffroyi	*Stenotrophomonas* sp.							-		-			+							+	+									-													
Artibeus fimbriatus	*Mammaliicoccus sciuri*						+		-	-						-				-													+			-		-					51
	Staphylococcus aureus						-		-	-						-				-													+			-		-					
	Staphylococcus saprophyticus						-		-	-						-				-													+			-		-					
	Staphylococcus kloosii						-		-	-						-				-													-			-		-					
	Stenotrophomonas sp.							-		-			+							+	+									-													49
	Escherichia coli	-	-	-		-		-	-	-			-		-					-	-																						
	Klebsiella oxytoca	-	-	+		-		-	-	-			-		-					-	-																						
	Serratia marcescens	+	-	+		+		-	-	-			-		-					-	-																						
Artibeus lituratus	*Enterobacter aerogenes*	+	-	+	-		+	+	-	-		-	-	+			-			-	-								-										-			-	52
	Erwinia herbicola	+	-	-	-		+	-	-	-		-	-	-			-			-	-								-										-			-	
	Kluyvera spp.	+	-	+	+		-	-	-	-		-	-	-			-			-	-								-										-			-	
	Koserella trabulsii	+	-	+	-		+	-	-	-		-	-	-			-			-	+								-										-			-	
	Proteus mirabilis	+	-	+	-		+	-	-	-		-	-	-			-			-	-								-										-			-	
	Serratia liquefaciens	+	-	+	-		+	-	-	-		-	-	-			-			-	-								-										-			-	
	Serratia rubidaea	+	-	+	-		+	-	-	-		-	-	-			-			-	-								-										-			-	
	Enterobacter agglomerans	+	-	+	-		+	-	-	-		-	-	-			-			-	-								-										-			-	
	Escherichia coli	+	-	+	+		+	-	-	-		-	-	+			+			+	-								+										+			-	
	Mammaliicoccus sciuri						+		-	-						-				-													+			-		-					51
	Staphylococcus aureus						-		-	-						-				-													+			-		-					
	Staphylococcus saprophyticus						-		-	-						-				-													+			-		-					
	Staphylococcus warneri						-		-	-						-				-													-			-		-					
	Staphylococcus kloosii						-		-	-						+				-													+			-		-					
	Salmonella sp.		-					-		-			-			-				-	-									-													49
	Escherichia coli	-	-	-		-		-	-	-			-		-					-	-																						
	Klebsiella oxytoca	-	-	+		-		-	-	-			-		-					-	-																						
	Serratia marcescens	+	-	+		+		-	-	-			-		-					-	-																						
Artibeus obscurus	*Stenotrophomonas* sp.							-		-			+							+	+									-													
	Escherichia coli	-	-	-		-		-	-	-			-		-					-	-																						
	Klebsiella oxytoca	+	-	+		+		-	-	-			-		-					-	-																						
	Serratia marcescens	+	-	+		+		-	-	-			-		-					-	-																						
Artibeus planirostris	*Enterobacter cloacae*	+	-	+	-		+	-	-	-		-	-	-			-			-	-								-										-			-	52
	Escherichia coli	+	-	+	-		-	-	-	-		-	-	-			-			-	-								+										+			-	
	Kluyvera spp.	+	-	+	-		-	-	-	-		-	-	-			-			-	-								-										-			-	
	Morganella morganii	+	-	+	-		-	-	-	-		-	-	-			-			-	-								-										-			-	
	Serratia liquefaciens	+	-	+	-		+	-	-	-		-	-	-			-			-	-								-										-			-	
	Enterobacter aerogenes	+	-	+	-		+	-	-	-		-	-	-			-			-	-								-										-			-	
	Enterobacter agglomerans	+	-	+	-		+	-	-	-		-	-	-			-			-	-								-										-			-	

	Erwinia herbicola	+	-	+	-		+	-	-	-		-	-	-			-			-	-								+										+			-	
Artebius sp.	*Escherichia coli*	-														+						+						-							+	-		-					36
Carollia perspicillata	*Mammaliicoccus sciuri*						+		-	-						-				-													+			-							51
	Pseudomonas auruginosa							-		-			-							-	-									-													49
	Stenotrophomonas maltophilia							-		-			+							+	+									-													
	Serratia marcescens	+	-	+		+		-	-	-			-		-					-	-																						
	Escherichia coli	-														+						-						-							-	-		+					36
	Escherichia coli	+	-	+	-		+	-	-	-		-	-	-			-			-	+								-										-			-	52
	Enterobacter aerogenes	+	-	+	-		+	-	-	-		-	-	-			-			-	-								-										-			-	
	Enterobacter agglomerans	+	-	+	+		+	+	+	-		+	+	+			+			-	+								-										-			-	
	Enterobacter cloacae	+	-	+	-		+	-	-	-		-	-	-			-			-	-								-										-			-	
	Erwinia herbicola	+	-	+	-		+	-	-	-		-	-	-			-			-	-								-										-			-	
	Proteus mirabilis	+	-	+	+		+	+	-	-		-	-	-			-			-	-								+										-			-	
	Serratia liquefaciens	+	-	+	-		-	-	-	-		-	-	-			-			-	-								-										-			-	
	Shigella sp.	+	+	+	+		+	+	-	-		+	+	+			+			+	+								-										-			-	
Cynopterus brachyotis javanicus	*Escherichia coli*																																			+							39
Cynopterus sphinx angulatus	*Escherichia coli*																																			+							
	Klebsiella pneumoniae			+																																							
	Klebsiella ozanae			+																																							
	Klebsiella aerogenes			+																																							
	Klebsiella oxytoca			+																																							
	Enterobacter aerogenes			+					+																											+		+					
	Enterobacter cloacae			+					+																											+		+					
Cynopterus tittaecheilus tittaecheilus	*Escherichia coli*																																			-							
	Klebsiella pneumoniae			+																																+		+					
	Klebsiella ozanae			+																																+		+					
	Klebsiella aerogenes			+																																+		+					
	Klebsiella oxytoca			+																																+		+					
	Enterobacter aerogenes			+																+																+		+					
	Enterobacter cloacae			+																+																+		+					
Desmodus rotundus	*Escherichia coli*	+	-		+	+	-	+	+	+		+		+			-	+		+	-		+					+	+			+		+		+			+	+	+		37
		+	-		+		-	+	+	+		+		+			-	+		+	-		+					+	+			+		+		+		+	-		+		46
		-														+						-						-							+	-		-					36
	Klebsiella oxytoca	-	-	+		-		-	-	-			-		-					-	-																						49
	Serratia marcescens	+	-	+		+		-	-	-			-		-					-	-																						
Dermanura cinerea	*Stenotrophomonas maltophilia*							-		-			+							+	+									-													
Diaemus youngi	*Escherichia coli*	-														+						-						-							+	-		+					36
Diphylla ecaudata	*Klebsiella oxytoca*	-	-	-		-		-	-	-			-		-					-	-																						49
	Serratia marcescens	+	-	+		+		-	-	-			-		-					-	-																						
Eidolon helvum	*Staphylococcus aureus*						-		-	+	+					+			+	-							-				-		+					-					53
											-					-		-		-			-								-		+			-		+					45
	Escherichia coli	+					-	-	+	+				-						+															+	+		+					35
	Staphylococcus schweitzeri										-					-		-		-			-								-		-			-		-					
	Staphylococcus argenteus										-					-		-		-			-								-		-			-		-					

Epomops franqueti	*Escherichia coli*	+	-	+	+	+	+	+		+		+		+		+	+	-		-		+	-					-	-			+		+	+	+		+	+		-		54
	Enterobacter cloacae	+	+	-	+	+	+	+		+		+		+		-	+	+		+		+	-					+	-			-		+	+	-		-	+		+		
	Klebsiella pneumoniae	+	-	-	+	+	+	+		+		+		+		-	+	+		+		+	+					-	+			+		+	+	+		+	+		+		
Eptesicus taddeii	*Staphylococcus aureus*																								+																		42
	Escherichia coli	+	+	-		+		-	-	-			-		-					-	-																						49
Eonycteris spelaea	*Waddlia malaysiensis*								-																								+		+			-					55
Glossophaga soricina	*Staphylococcus epidermidis*						-		-	-						-				-													+			-		-					51
	Staphylococcus xylosus						-		-	-						-				-													+			-		-					
	Staphylococcus aureus						-		-	-						-				-													+			-		-					
Glossophaga sp.	*Escherichia coli*	-														+						-						-							-	-		-					36
Glyphonycteris sylvestris	*Serratia marcescens*	+	-	+		+		-	-	-			-		-					-	-																						49
Hipposideros larvatus	*Mammaliicoccus sciuri*						-		-	-	+					-			+	-							-						-			-		-					56
	Salmonella gallinarum						-		-	-	+					-			+	-							-						-			-		-					
	Staphylococcus nepalensis						-		-	-	+					-			-	-							-						-			-		-					
Histiotus macrotus	*Rahnella aquatilis*	+		+		+			+					+				+															+	+					+	+			40
Histiotus velatus	*Serratia marcescens*	+	-	+		+		-	-	-			-		-					-	-																						49
Hypsignathus monstrosus	*Staphylococcus aureus*																								+																		42
Lasiurus cinerus	*Rahnella aquatilis*	+		+		+			+					+				+															+	+					+	+			40
Lasiurus ebenus	*Serratia marcescens*	+	-	+		+		-	-	-			-		-					-	-																						49
	Pseudomonas auruginosa							-		-			-			-				-	-									-													
Lasiurus varius	*Rahnella aquatilis*	+		+		+			+					+				+															+	+					+	+			40
Megaloglossus woermann	*Escherichia coli*	+	+	+	+	+	+	+		+		+		+		+	+	+		+		+	+					+	+			+		+	+	+		+	+		+		54
	Klebsiella pneumoniae	+	-	-	+	+	+	+		+		+		+		-	-	-		+		+	-					+	+			-		+	-	+		+	+		+		
Micronycteris microtis	*Serratia marcescens*	+	-	+		+		-	-	-			-		-					-	-																						49
Mimon bennetti	*Klebsiella oxytoca*	-	-	-		-		-	-	-			-		-					-	-																						
	Serratia marcescens	+	-	+		+		-	-	-			-		-					-	-																						
Molossus cf. currentium	*Serratia marcescens*	+	-	+		+		-	-	-			-		-					-	-																						
Molossus major	*Escherichia coli*	-														+						-						-							-	-		+					36
Molossus molossus		-	-	-		-		-	-	-			-		-					-	-																						49
	Klebsiella oxytoca	-	-	+		-		-	-	-			-		-					-	-																						
Molossus neglectus	*Escherichia coli*	+	+	-		+		-	-	-			-		-					-	-																						
	Serratia marcescens	-	-	-		+		-	-	-			-		-					-	-																						
Molossus rufus	*Acinetobacter baumannii*		-	-		-		-	-	+			-							-	-									-													
	Salmonella sp.	-	-					-	-	-			-							-	-																						
	Escherichia coli	-	-	+		+		-	-	-			-		-					-	-																						
	Serratia marcescens	+	-	+		+		-	-	-			-		-					-	-																						
Mormoops sp.	*Escherichia coli*	-														+						-						-							-	-		-					36
Myonycteris torquata	*Escherichia albertii*	-	-	-		-			-											-																-		-					43
Myotis myotis	*Arthrobacter* sp.			+					+																																	+	57
	Kocuria sp.								+																																	+	
Myotis nigricans	*Serratia marcescens*	+	-	+		+		-	-	-			-		-					-	-																						49
Myotis ruber	*Serratia marcescens*	+	-	+		+		-	-	-			-		-					-	-																						
Noctilio leporinus	*Escherichia coli*	-														+						-						-							-	-		+					36

Nycteris hispida	*Escherichia coli*	+					-	+	+	+				+						+															+	+		+					61
Phyllostomus discolor	*Escherichia coli*	-														+						-						-							+	-		+					36
Phyllostomus hastatus	*Escherichia coli*	+														+						-						+							+	+		+					
Platyrrhinus lineatus	*Staphylococcus nepalensis*						-		-	+						+				-													-			-		-					51
	Staphylococcus haemolyticus						+		-	-						+				-													+			-		-					
	Klebsiella oxytoca	-	-	+		-		-	-	-			-		-					-	-																						49
Platyrrhinus recifinus	*Staphylococcus aureus*						-		-	-						-				-													+			-		-					51
	Serratia marcescens	+	-	+		+		-	-	-			-		-					-	-																						49
Pteronotus parnelli	*Escherichia coli*	+														+						+						-							+	-		-					36
Pteropus poliocephalus	*Escherichia coli*	+		+		+			+	+				+							+							+							+	+		+					58
		+																																									44
Pygoderma bilabiatum	*Mammaliicoccus sciuri*						-		-	-						-				-													+			-							51
	Stenotrophomonas sp.							-		-			+							-	+									-													49
	Serratia marcescens	+	-	+		+		-	-	-			-		-					-	-																						
	Escherichia coli	-	-	+		-		-	-	-			-		-					-	-																						
Rhinolophus hipposideros	*Moellerella* spp.	+		+																+	+							+							+						+		38
	Staphylococcus nepalensis																																									+	57
Rhinolophus monoceros	*Bacillus cereus*	-					+		-	-			-	-																		-	+	-				-				-	12
	Bacillus anthracis	-					+		-	+			-	-																		-	-	-				+				-	
	Pseudomonas stutzeri	-					-		-	-			-	-																		-	-	-				-				-	
Sturnira lilium	*Enterobacter aerogenes*	+	-	+	-		+	-	-	-		-	-	+			-			-	-								-										-			-	52
	Enterobacter agglomerans	+	-	+	-		+	-	-	-		-	-	-			-			-	-								-										-			-	
	Serratia liquefaciens	+	-	+	-		+	-	-	-		-	-	-			-			-	-								-										-			-	
	Ewingella americana	+	-	-	-		+	-	-	-		-	+	-			-			-	-								-										-			-	
	Escherichia coli	+	-	+	-		+	-	-	-		-	-	+			-			-	-								-										-			-	
	Mammaliicoccus sciuri						+		-	-						+				-													-			-		-					51
	Staphylococcus aureus						-		-	-						+				-													+			-		-					
	Staphylococcus warneri						-		-	-						-				-													-			-		-					
	Staphylococcus xylosus						-		-	-						-				-													+			-		-					
	Escherichia coli	-	-	-		-		-	-	-			-		-					-	-																						49
	Serratia marcescens	+	-	+		+		-	-	-			-		-					-	-																						
Sturnira tildae	*Stenotrophomonas* sp.							-		-			+							+	+									-													
	Escherichia coli	-	-	-		-		-	-	-			-		-					-	-																						
Tadarida teniotis	*Staphylococcus* spp.						-		-	-	-					+			-	-				-			-								+	-	-	-			-		47
	Escherichia coli	+	+	+										+														+							+	+		+					48
Tadarida brasilensis	*Rahnella aquatilis*	+		+		+			+					+				+															+	+					+	+			40
Trachops cirrhosus	*Escherichia coli*	-	-	-		-		-	-	-			-		-					-	-																						49
	Serratia marcescens	+	-	+		+		-	-	-			-		-					-	-																						
**Miniopterus schreibersii / Myotis myotis / Rhinolophus hipposideros*	*Pseudomonas* spp.	+		+	+			+	+					x						+	+					+		+							+	+		+			+		38
	Enterobacter spp.	+		+	+			+	+					x						+	+					+		+							+	+		+			+		
	Proteus spp.	+		+				+						+						+						+									+			+			+		

*Myotis capaccinii / Miniopterus schreibersii / Myotis myotis / Rhinolophus hipposideros	*Klebsiella* spp.	+		+	+			+						x						+	+							+							+	+					+		
	Citrobacter spp.	+		+	+			+						x						+	+					+		+							+			+			+		
	Serratia spp.	+		+	+			+						x						+	+					+		+							+			+			+		
	Hafnia spp.	+		+	+			+						x						+	+							+							+						+		
	Providencia spp.	+		+	+			+						x						+	+					+		+							+			+			+		
	Morganella spp.	+		+																+	+					+									+			+			+		
	Staphylococcus spp.	+		+				+						+		+				+	+			+		+									+			+			+	+	
	Bacillus spp.	+		+				+						+		+				+	+			+		+									+			+			+	+	
*Miniopterus schreibersii / Rhinolophus hipposideros	*Achromobacter* spp.	+		+	+			+	+					+						+															+						+		
	Proteus spp.	+		+				+						+						+	+					+									+			+			+		
	Alcaligenes spp.	+		+				+						+						+															+						+		
*Miniopterus schreibersii / Myotis myotis	*Kluyvera* spp.	+		+	+															+								+							+						+		
	Enterococcus spp.	+		+				+						+		+				+	+			+		+									+			+			+	+	
	Aerococcus spp.							+						+						+															+						+		
*Eidolon helvum, Epomops franqueti, Hypsignathus monstrosus, Myonycteris torquata, Rousettus aegyptiacus	*Escherichia coli*	-	-	-		-			-											-																-		-					43
*Plecotus auratus, Myotis daubentonii	*Escherichia coli*			+				-	+	+				+						+		+						+							+	+		+					3
*Miniopterus schreibersii, Myotis myotis, Myotis blythii and Myotis capaciinii	*Escherichia coli*			-					-	+																		+								+		-					59
*Myotis myotis, Myotis blythii	*Staphylococcus nepalensis*			-					-							-				-		-					-								-		-	-				-	60

A: amoxicillin, Am: Amikacin, Ap: Ampicillin, Az: Aztreonam, C: Cephalexin, Ce: Cefoxitin, Cf: Ceftazidime, Ch: Chloramphenicol, Ci: Ciprofloxacin, Cl: Clindamycin, Cp: Cefepime, Ct: ceftriaxone, Cx: Cefotaxime, Do: doxycycline, E: erythromycin, Er: Ertapeneme, Fo: Fosfomycin, Fu: Fusidic Acid, Ge: Gentamicin, I: Imipeneme, K: kanamycin, Le: Levofloxacin, Li: lincomycin, M: Methicillin, Mi: Minocycline, Mu: Mupirocin, N: nalidixic acid, Ne: Netilmicin, No: norfloxacin, O: Oxacillin, Of: Ofloxacin, Pe: Penicilin, Pi: Piperacillin, S: streptomycin, Sx: sulphamethoxazole/trimethoprim, Tc: teicoplanin, TE: tetracycline, Ti: Ticarcillin, Tm: Temocilin, To: tobramycin, Va: vancomycin, +: resistance, -: susceptible, So: Source, *: publication show AMR in several bat species but there is no way to know in which one.
